# Semi-natural housing rescues social behavior and reduces repetitive exploratory behavior of BTBR autistic-like mice

**DOI:** 10.1038/s41598-023-43558-0

**Published:** 2023-09-27

**Authors:** Matthew S. Binder, Angelique Bordey

**Affiliations:** Departments of Neurosurgery, and Cellular & Molecular Physiology, Wu Tsai Institute, Yale University School of Medicine, 333 Cedar Street, New Haven, CT 06520-8082 USA

**Keywords:** Neuroscience, Diseases

## Abstract

Environmental enrichment confers numerous benefits when implemented in murine models and can reduce behavioral symptomatology in models of disease, such as autism spectrum disorder (ASD). However, previous work did not examine the impact of early-life environmental enrichment on each core feature of ASD. We thus implemented a social and physical enrichment at birth, modeling a semi-natural housing, and examined its impact on communicative, social, sensory, and repetitive behaviors using BTBR (autistic-like) and C57BL/6 J (B6, wildtype) mice, comparing them to standard housing conditions. We found that environmental enrichment alleviated the social deficit of juvenile BTBR mice and reduced their repetitive exploratory behavior but did not affect their rough versus smooth texture preference nor the number of maternal isolation-induced pup calls. Environmental enrichment only affected the call characteristics of B6 mice. One interpretation of these data is that early-life environmental enrichment has significant therapeutic potential to treat selective core features of ASD. Another interpretation is that reducing environmental complexity causes selective behavioral deficits in ASD-prone mice suggesting that current standard housing may be suboptimal. Overall, our data illustrate the extent to which the environment influences behavior and highlights the importance of considering housing conditions when designing experiments and interpreting behavioral results.

## Introduction

Autism Spectrum Disorder (ASD) is a prevalent neurodevelopmental condition that is characterized by deficits in sociability and communication, stereotypy, and abnormal sensory processing^1^. Pharmaceutical treatment options have thus far showed limited efficacy to decrease symptom severity of ASD, however, behavioral treatments have shown some success. One particularly effective and low-cost option is environmental enrichment^2,3^. Daily sensory enrichment therapy implemented in children with ASD improved attention span, motor skills, sensory processing, self-awareness, communication, and social skills^2^. Furthermore, early intensive behavioral intervention (EBI) implemented in children with ASD improved social behavior^3^. While these studies demonstrate that modifying the environment can have profound effects on the autistic phenotype, the corresponding underlying mechanisms remain ambiguous.

Environmental enrichment in autistic-like murine models can help to clarify its underlying mechanisms and potential efficacy. In murine models, environmental enrichment typically refers to either physical enrichment, wherein the animals are placed in a much larger cage that has objects designed to stimulate curiosity, or social enrichment, wherein animals from different litters are either reared together or allowed to interact^4–6^. Environmental enrichment in neurotypical mice has been shown to improve learning and memory, decrease anxiety, and contribute to beneficial morphological changes such as increased dendritic branching and cortical thickness^7–9^. In ASD mouse models, environmental enrichment has been shown to selectively increase social behaviors and to reduce stereotypy^10–12^, as observed in humans^2,3^. While these results suggest that the effects of environmental enrichment are conserved across species, most studies have typically focused on learning and memory, sociability, anxiety, and repetitive behaviors, therefore the effects of environment on other core ASD phenotypes, such as communicative and sensory behaviors, are unknown. In addition, nearly all studies implemented environmental enrichment in adult animals. Since ASD is typically diagnosed early in life and is a disorder of brain development, it is important to examine the impact of enrichment soon after birth.

To elucidate the effects of early life environmental enrichment on the autistic phenotype, the current study implemented environmental enrichment at birth to model semi-natural housing. We used BTBR *T*^+^ *Itpr3*^*tf*^/J (BTBR), autistic-like, mice and assessed core features of ASD: communication, sociability, repetitive behavior, and tactile sensory preference. C57BL/6 J (B6) mice were also assessed to contextualize any improvement in the BTBR mice and to elucidate the effects of early life environmental enrichment in wildtype mice. Collectively, our study is the first to comprehensively assess the effects of semi-natural housing on each aspect of autistic-like behavior and is one of the few studies to implement enrichment at birth, helping to clarify the potential therapeutic effects of environmental enrichment in an autistic model.

## Results

**Figure 1:**
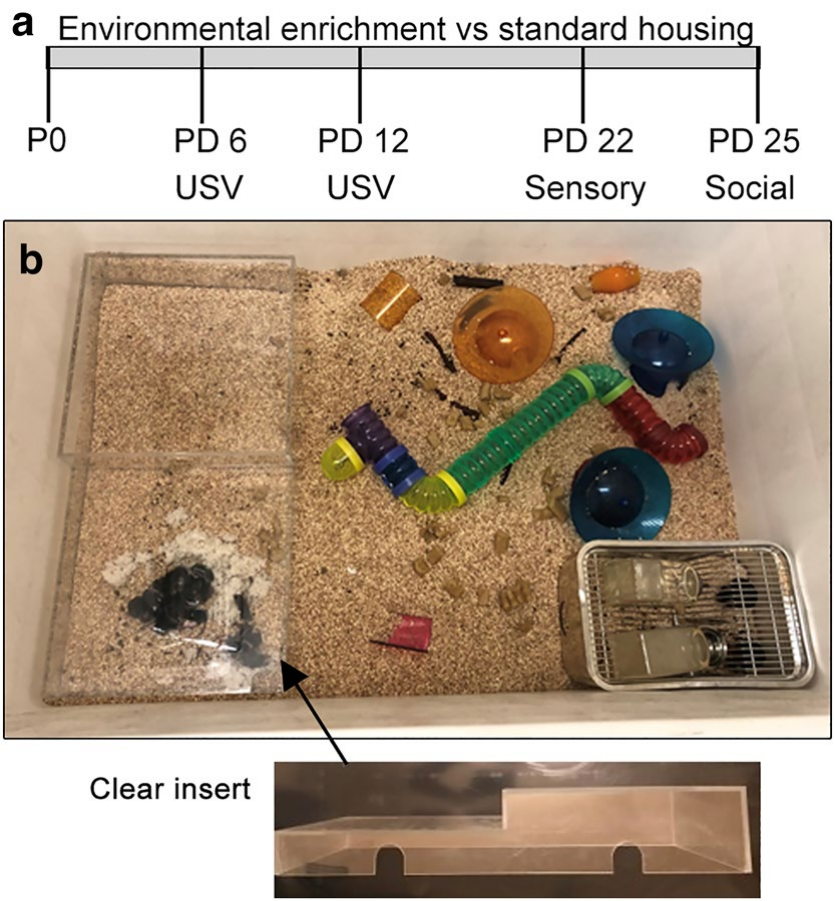
Experimental design and housing. (**a**) Depiction of the experimental timeline. (**b**) Images of the housing with physical and social environmental enrichment.

The Fig. 1 illustrates the overall experimental paradigm and the semi-natural housing that includes both social and physical enrichment for BTBR and B6 mice. Two dams of the same genotype and their respective litters (at birth) were placed into the semi-natural housing at a time. Mice in the control housing condition were raised in a standard cage with one litter per cage. Upon being placed in the enriched housing, the dams immediately made a nest out of the available material inside of the custom housing unit and then moved the pups into the unit. The pups slept together in the custom housing unit and were not seen venturing out for the first two weeks of life. Further observations revealed that the maternal behavior was similar between BTBR and B6 mice, with the dams alternating caring for the pups. Both dams brought food back to and stored it in the housing unit. Around postnatal day (PD) 16, some pups would wander near and around the housing unit and by PD 18-PD 20 pups would wander extensively around the enclosure, however, they tended to congregate in two enclosed spaces: in the custom housing area and in the tube in the center of the arena. Mice were not observed running on the wheels during the periodic checks in the light cycle and little fecal matter or debris was found on them, suggesting minimal use at night. Since no differences were found between males and females for any behavior in both strains of mice, data from males and females were combined into one group (Tables S1, S2, S3).

## Ultrasonic vocalizations

**Figure 2:**
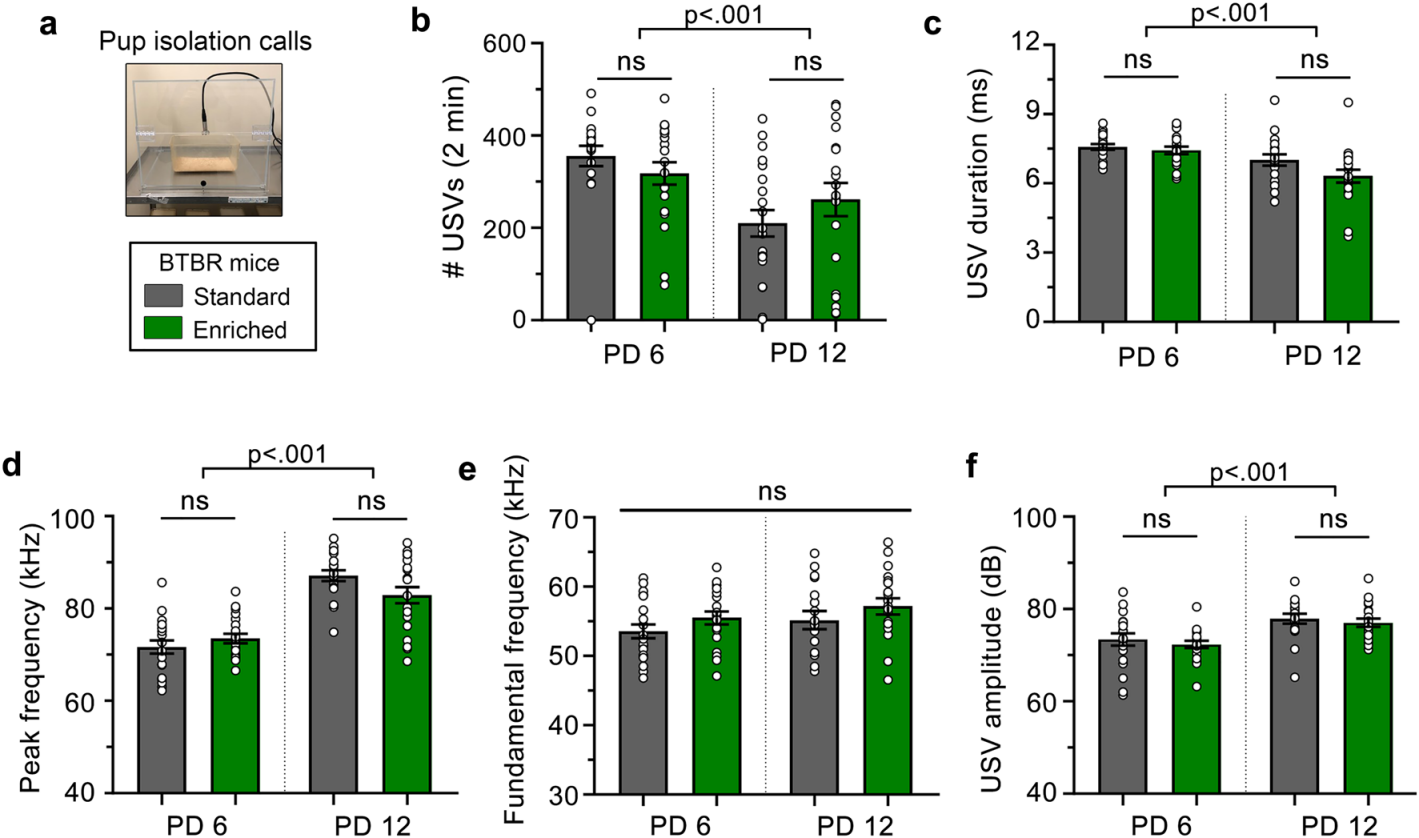
BTBR maternal isolation-induced USVs. (**a**) Diagram of the experimental design. (**b**) The number (#) of USVs were not affected by the environmental enrichment at either age, but decreased from PD 6 to PD 12 in both conditions (Mixed effects ANOVA: condition (standard vs enriched): F(1, 36) = 0.05, *p* = 0.83, condition x day: F(1, 36) = 2.78, *p* = 0.10, day: F(1, 36) = 14.45, *p* < 0.001). (**c**) USV duration was not affected by environmental enrichment at either age, but decreased over time in both conditions (Mixed effects ANOVA: condition: F(1, 33) = 3.48, *p* = 0.07, condition x day: F(1, 33) = 2.05, *p* = 0.16, day: F(1, 33) = 18.31, *p* < 0.001). (**d**) Peak frequencies were not affected by the housing condition at either time point, but were increased across time in both conditions (Mixed effects ANOVA: condition: F(1, 33) = .29, *p* = 0.59, condition x day: F(1, 33) = 6.93, *p* = 0.01, and day: F(1, 33) = 103.4, *p* < 0.001). (**e**) Fundamental frequencies were not affected by any variables (Mixed effects ANOVA: condition: F(1, 33) = 2.57, *p* = 0.12), condition x day: F(1, 33) = 0.00, *p* = 0.98, day: F(1, 33) = 2.62, *p* = 0.12). (**f**) Amplitudes were not affected by the housing condition at either time point, but were increased across time (Mixed effects ANOVA: condition: F(1, 33) = 0.59, *p* = 0.45, condition x day, *p* = 0.99, day: F(1, 33) 22.76, *p* < 0.001). For every graph, the listed p values were obtained with Sidak post-hoc tests. ns: not significant.

To assess the impact of ASD genes on an early form of social communication, many studies have used a well-conserved behavioral paradigm in mice, the neonatal isolation paradigm, since both human infants and mouse pups cry atypically when they are physically separated from their mothers^13,14^. In addition, an infant’s cry is a form of evolutionarily conserved social communication that is altered in mouse models of ASD^15,16^ and has been proposed as an early sign of ASD^17^. BTBR mice have been reported to display alterations in maternal isolation-induced pup calls or ultrasonic vocalizations (USV)^18^. For BTBR pups, the environment (enriched vs standard) did not impact any characteristics of the USVs at PD 6 and PD 12 (Fig. 2). These characteristics include production (i.e., number of calls), duration, peak and fundamental frequency (i.e., pitch), and amplitude (i.e., loudness) of USVs (ANOVA statistics and post-hoc p values are listed in the figure legends) (Fig. 2b,c,d,e,f). However, the following characteristics of USVs significantly changed from PD 6 to PD 12 in both housing conditions: decreased number, decreased duration, higher peak frequency, and increased amplitudes, while there was no change in fundamental frequency (Fig. 2b,c,d,e,f).

**Figure 3:**
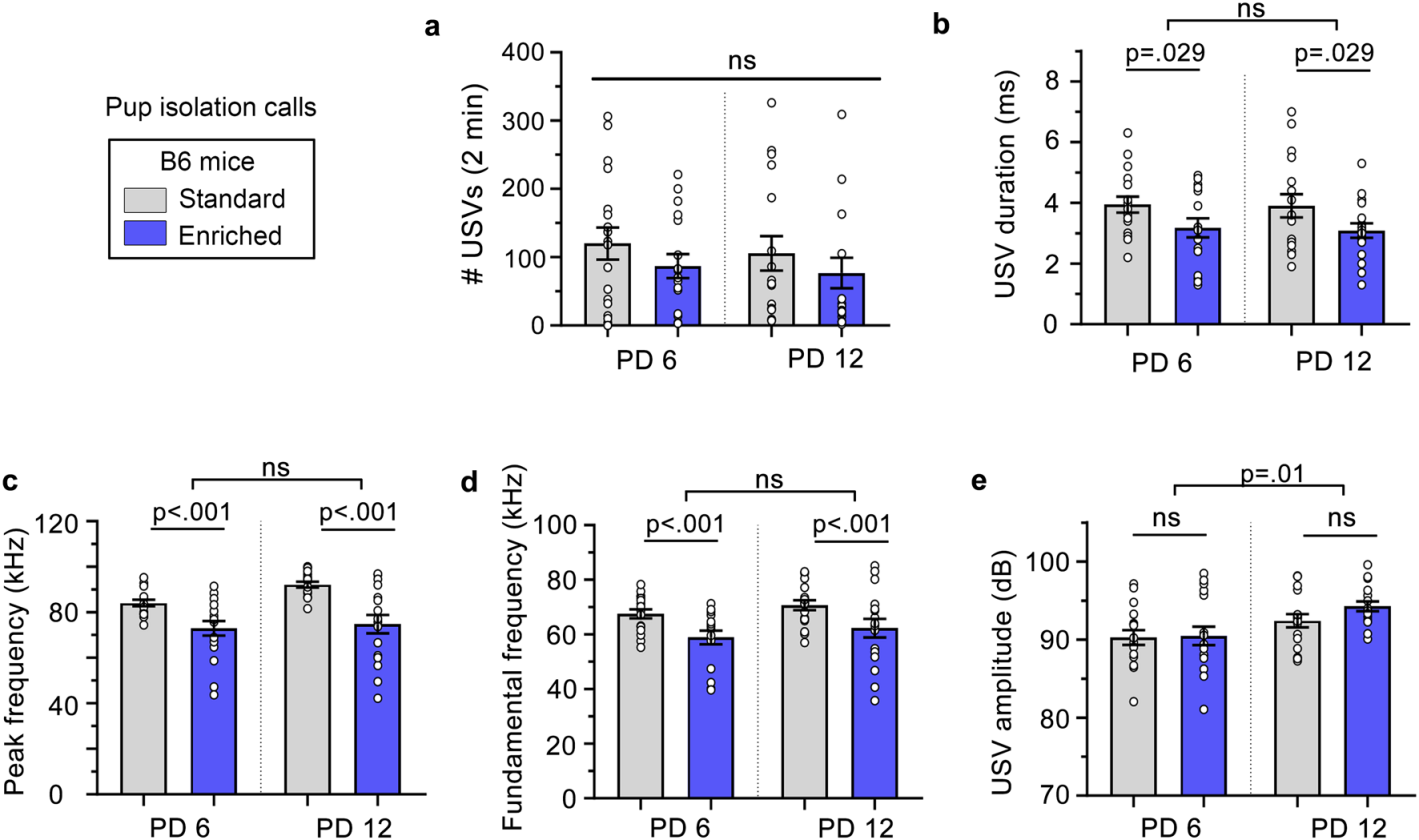
Maternal isolation-induced USVs of B6 pups. (**a**) The number (#) of USVs was not affected by either housing condition or age (Mixed effects ANOVA: condition: F(1, 30) = 1.47, *p* = 0.24, condition x day: F(1, 30) = 0.02, *p* = 0.90, and day: F(1, 30) = 0.59, *p* = 0.45). (**b**) USV’s durations were longer in the standard versus enriched housing group but were not affected by age (Mixed effects ANOVA: condition: F(1, 29) = 5.38, *p* = 0.03, condition x day: F(1, 29) = 0.006, *p* = 0.94, day: F(1, 29) = 0.20, *p* = 0.66). (**c**, **d**) The peak and fundamental frequencies were higher in the standard vs enriched housing condition but were not affected by age (Mixed effects ANOVA: peak frequency: condition: F(1, 29) = 31.81, *p* < 0.001, condition x day: F(1, 29) = 1.22, *p* = 0.28, day: F(1, 29) = 2.67, *p* = 0.11), (fundamental frequency: condition: F(1, 29) = 16.17, *p* < 0.001, group x day F(1, 29) = 0.00, *p* = 0.98, day F(1, 29) = 1.60, *p* 0.22). (**e**) USV’s amplitudes (i.e., loudness) were not affected by the housing condition, but increased with age (Mixed effects ANOVA: condition: F(1, 29) = 1.88, *p* = 0.18, condition x day: F(1, 29) = 0.87, *p* = 0.36, day: F(1, 29) = 6.90, = 0.01). For every graph, the listed p values were obtained with Sidak post-hoc tests. ns: not significant.

In B6 pups, USV production and amplitudes were not affected by the enriched environment (Fig. 3a,e), but the duration, and peak and fundamental frequencies were decreased by environmental enrichment (Fig. 3b,c,d). For most USV characteristics, except the amplitude, there were no differences between PD 6 and PD 12. For USV amplitudes, pups emitted louder calls at PD 12 vs PD 6 (Fig. 3e). Overall, early environmental enrichment using a semi-natural housing had some effects on the specific characteristics of calls in B6 mice but had no effect on the number of BTBR and B6 calls and on the loudness of B6 calls.

### Three chamber sociability assessment

**Figure 4:**
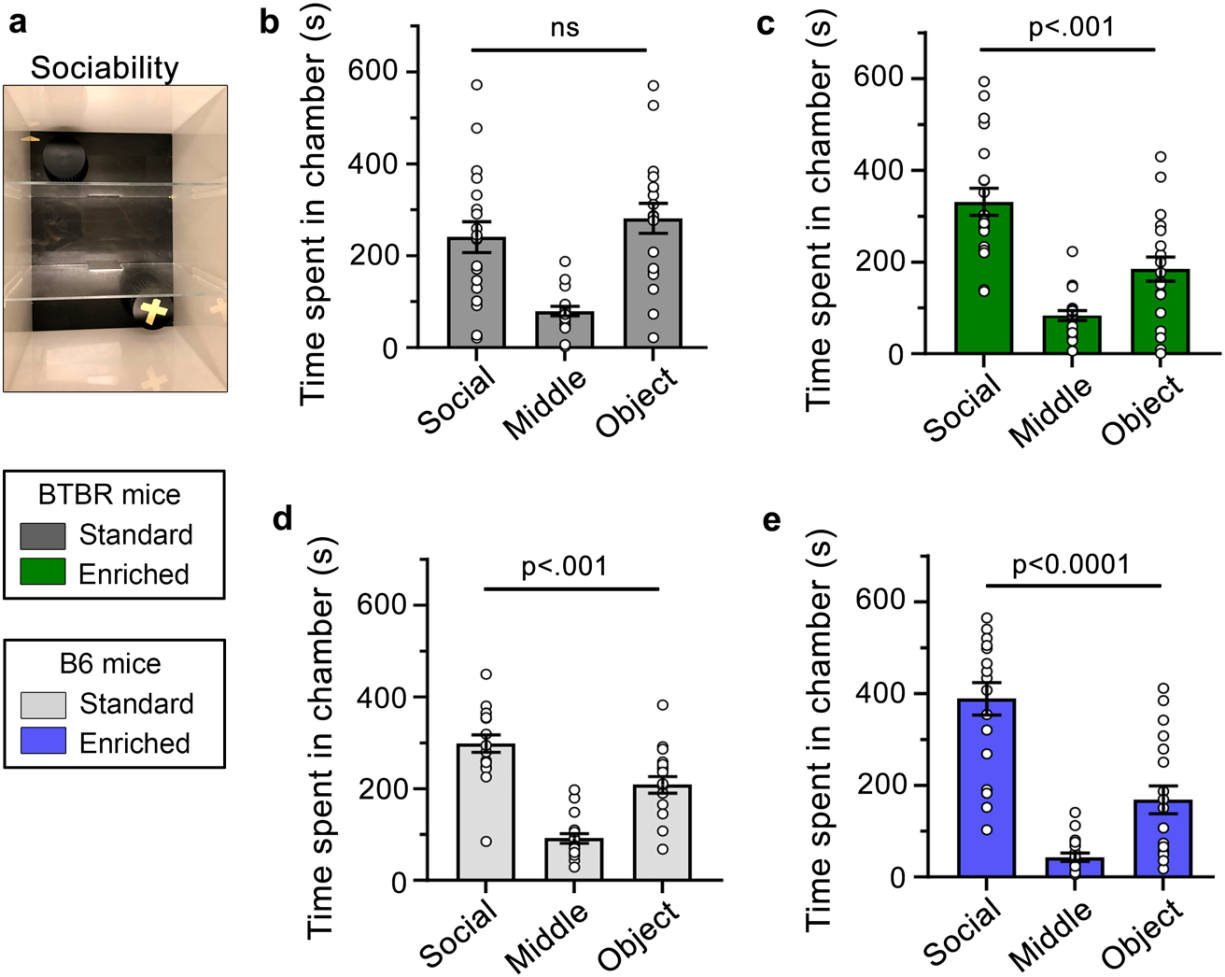
The effects of environmental enrichment on social behavior. (**a**) Image of the three-chamber social paradigm. (**b**) Standard housing BTBR mice spent a similar amount of time in the social and object chambers and less time in the middle chamber (One-way ANOVA: F(2, 54) = 14.89, *p* < 0.0001). (**c**) BTBR mice in the enriched condition spent more time in the social chamber than the object chamber and spent the least time in the center (One-way ANOVA: F(2, 57) = 27.80, *p* < 0.0001). (**d**, **e**) B6 mice in both the standard (**d**) and enriched (**e**) conditions spent more time in the social chamber than the object chamber and spent the least time in the center (One-way ANOVA: (**d**) F(2, 51) = 40.53, *p* < 0.0001 and (**e**) F(2, 51) = 40.43, *p* < 0.0001). For every graph, the listed p values were obtained with Tukey post-hoc tests -comparison values between the social and object chambers are depicted. ns: not significant.

We analyzed the impact of the semi-natural housing on the social behavior of BTBR and B6 juvenile mice (PD 25) using the classic three-chamber sociability task (Fig. 4a)^19^. BTBR mice raised in standard housing spent a similar amount of time in the chamber with a stranger mouse (called social chamber) as they did in a chamber with an object (called object chamber), displaying a pronounced social deficit (Fig. 4b), as previously reported^20,21^. However, BTBR mice raised in an enriched environment spent significantly more time in the social chamber than the object chamber (Fig. 4c), suggesting that early environmental enrichment corrected the social deficit of BTBR mice. B6 mice raised in either standard or enriched housing spent significantly more time in the social chamber than in the object chamber (Fig. 4d,e). Overall, early life enrichment increases social behavior in ASD-like animals.

### Tactile sensory preference assessment

**Figure 5:**
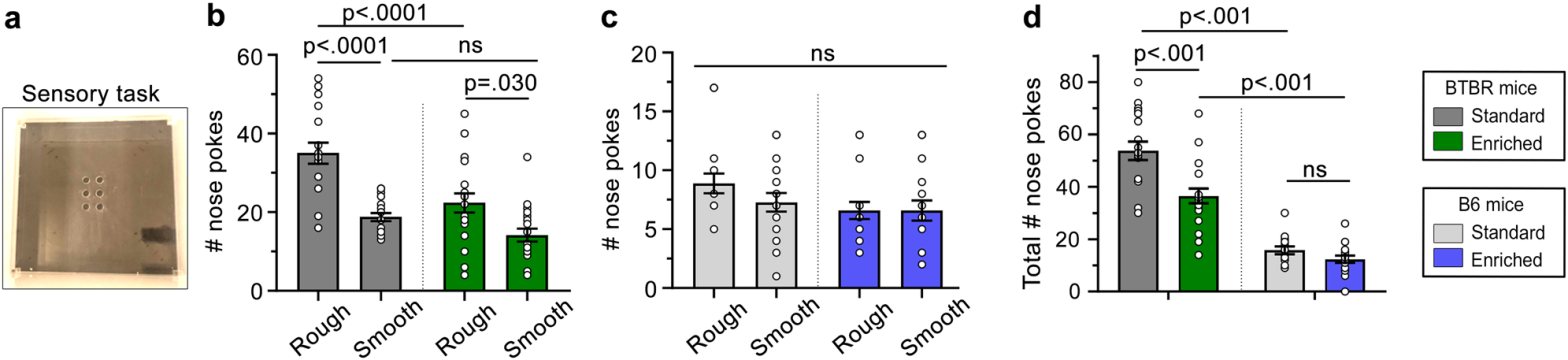
The effects of environmental enrichment on tactile sensory preferences and exploration of novel objects. (**a**) Image of the SNAP apparatus. (**b**) BTBR mice produced more rough than smooth nose pokes in both housing conditions although enriched mice produced fewer rough nose pokes than standard housed animals (Mixed effects ANOVA: condition: F(1, 36) = 14.10, *p* < 0.001, texture: F(1, 36) = 42.33, *p* < 0.001, texture x condition: F(1, 36) = 4.59, *p* = 0.04). (**c**) The number of nose pokes by B6 mice were not affected by the housing condition or the texture (Mixed effects ANOVA: housing: F(1, 30) = 1.15, *p* = 0.29, texture: F(1, 30) = 3.15, *p* = 0.09, texture x group F(1, 30) = 1.89, *p* = 0.18). ns: not significant. (**d**) BTBR mice in standard and enriched housing produced more nose pokes than B6 mice and more nose pokes in the standard vs the enriched condition while the housing conditions did not affect the number of nose pokes by B6 mice (Two-way ANOVA: condition: F(1, 75) = 17.32, *p* < 0.001, strain: F(1, 75) = 156.11, *p* < 0.001, strain x condition: F(1, 75) = 7.72, *p* = 0.007). For every graph, the listed p values were obtained with Sidak (**b** and **c**) and Tukey (**d**) post-hoc tests. ns: not significant.

Tactile sensory preference and repetitive exploration of novel objects were assessed for juvenile mice (PD 22) using one newly developed behavioral task, the novel somatosensory nose-poke adapted paradigm (SNAP) wherein mice have the option of poking their noses into smooth or rough holes (Fig. 5a)^22^. Independent of the housing conditions, BTBR mice produced more rough hole pokes than smooth hole pokes, suggesting that BTBR mice prefer rough surfaces (Fig. 5b). However, BTBR mice raised in the enriched condition produced significantly fewer rough hole pokes than those raised in the standard condition (Fig. 5b). By contrast, B6 mice showed no preferences for smooth or rough holes in each housing condition (Fig. 5c).

To assess repetitive exploratory behavior in BTBR mice, we summed the total number of nose pokes in both smooth and textured holes (Fig. 5d). Independent of the housing conditions, BTBR performed more total nose pokes than B6 mice, suggesting that they display increased exploration of novel objects, or in other terms, a repetitive exploratory behavior compared to B6 mice. The enriched environment reduced, although did not rescue, the total number of nose pokes performed by BTBR mice. By contrast, the enriched environment did not affect the exploratory behavior (total number of nose pokes) of B6 mice. Collectively, these data suggest that being raised in semi-natural housing reduced the repetitive exploratory behavior of BTBR mice but did not attenuate their rough texture preference.

## Discussion

Our study examined the effects of environmental enrichment, using a semi-natural housing implemented at birth, on communicative, social, repetitive exploratory, and sensory behaviors of an ASD and a control mouse model. Like prior studies, we found a pronounced social deficit in BTBR mice raised in standard housing^20,21^. Importantly, raising BTBR animals in an enriched environment was sufficient to change these animals' social behavior, rendering them indistinguishable from prosocial, B6 mice. Queen et al. (2020) assessed environmental enrichment in adult BTBR mice and found an increase in male but not female sociability. Since we did not find any sex specific changes in our study, this suggests that there is a critical period for improving social behavior in female mice. The prosocial effects of environmental enrichment have also been found in 3–4-week-old female *Pten* heterozygous mice, another ASD mouse model^10^. This suggest that the beneficial effects of environmental changes on social behavior, a core ASD feature, is conserved across several ASD mouse models.

The beneficial effects of environmental enrichment were not limited to only social behaviors but also extended to repetitive exploratory behavior. Specifically, BTBR mice raised in standard or enriched housing produced significantly more nose pokes than control animals, suggesting a repetitive exploratory behavior compared to B6 mice. This suggests either an increased stereotypy or interest in novel objects or a combination of both. BTBR mice have been shown to have increased stereotypy^23^. When raised in a semi-natural housing, BTBR mice displayed a reduction in repetitive exploratory behavior. Such an effect of environmental enrichment has been reported in both ASD models and in control (wildtype) mouse models, indicating that these effects are not strain-nor disease state-dependent^10,24,25^. Collectively, these studies highlight the striking degree to which mice are sensitive to environmental changes, as simply increasing environmental complexity is sufficient to rescue social behavior and decrease repetitive exploratory behaviors of ASD-like mice.

To get a more comprehensive understanding of the effects of environmental enrichment, we broadened our assessment to include communication and sensory behaviors, two previously unassessed phenotypes. We found that BTBR mice displayed an abnormal pattern of USV production as previously reported^18^. However, environmental enrichment did not affect vocal production in BTBR or B6 mice. We also assessed tactile sensory preference as the new DSM-V definition of ASD includes sensory issues as one of the four restricted/repetitive behavioral features defined as: “hyper or hypo reactivity to sensory input or unusual interest in sensory aspects of the environment^1^”. Furthermore, sensory abnormalities are understudied in ASD despite their high prevalence (90% of all cases)^26^. BTBR mice displayed a strong behavioral sensory preference towards rough textures whereas B6 mice showed no texture preference. Environmental enrichment did not correct abnormal sensory behavior of BTBR mice, nor did it impact sensory behavior in B6 mice.

Our study has two major differences compared to previous studies using environmental enrichment. One difference is that BTBR mice were placed in an enriched environment starting at birth whereas other studies subjected juveniles or adults to environmental enrichment prior to assessing ASD behavior. Another difference is that we maximized environmental enrichment to assess its highest net effect while other studies focused on social (2 litters), environmental (toys and hide-outs), or physical (exercise) enrichment independently–not concurrently. These differences may explain the strong impact we observed on social and repetitive behaviors in our study compared to the smaller impact found in the other studies^11,27,28^. However, one disadvantage of our study is that it is difficult to parse out which type of enrichment had the greatest effects on the assessed behavior. Regarding exercise, mice were in a large cage and had access to running wheels but a study that assessed the effects of voluntary exercise in BTBR mice found no effect on sociability^28^. Mice slept together in one huddle providing more physical contact. Another limitation is that we did not monitor animal behavior during the night cycle and cannot provide observations regarding their level of activity and social interactions (e.g., play). Thus, determining the precise contribution of each form of enrichment on behavior is an important future direction of research and would provide a more comprehensive understanding of the relationship between environment and behavioral phenotypes.

One outcome of our findings is that early environmental enrichment can have significant therapeutic value, particularly for social and repetitive behaviors. Our study compliments and extends the existing literature and suggests that there may be a critical window wherein environmental enrichment yields the greatest effects. Another outcome is that the terms “standard housing” or “enriched environment” need to be reassessed. Wild mice live in dynamic and complex environments that better resemble enriched conditions than the standard (control) housing conditions, which are impoverished by comparison. Similarly, an enriched housing for mouse models would better reflect the complex environment and lifestyles of humans. A reliance upon standardized environmental impoverishment may have long reaching implications for behavior. For instance, we found that environmental enrichment reverses a social deficit in an ASD mouse model. These data suggest that as housing environments become less complex (more impoverished), there is an increase in behavioral deficits–at least in a disease model. Thus, decreased environmental complexity may exaggerate or even create deficits (behavioral artifacts) that can lead to misinterpretations, therefore enriching the environment may simply correct a behavioral artifact and not a true phenotypic feature of a model. Altogether, there is compelling evidence to suggest that an “enriched” housing environment may lead to fewer behavioral artifacts and therefore should become the standard housing option.

In conclusion, future studies need to be conducted to determine what constitutes enrichment and a reliable housing baseline. Studies could also investigate how to optimize enrichment so it more easily implementable in a vivarium setting. Altogether, this work is crucial as determining the scope and breadth of environmental enrichment on key autistic features in humans is contingent on having a valid proxy in animal models.

## Methods

### Animals

C57BL/6 J (B6) and BTBR mice were purchased from Jackson laboratories. Overall, 75 mice were used. Specifically, for B6 mice, 17 (9 males, 8 females) mice were reared in an enriched environment while 18 (9 males, 9 females) mice were reared in a standard housing environment, totaling 35 animals. For BTBR (autistic-like) mice, 20 (10 male, 10 female), mice were raised in an enriched environment and 20 (10 male, 10 female) mice were raised in a standard housing environment, totaling 40 animals. One BTBR and one B6 mouse were excluded from the three-chamber assessment due to computer error. Animals were tested during the light cycle, between 1 and 4 p.m. Furthermore, all behavioral testing was done prior to weaning to avoid incest. Mice were housed in a climate-controlled room on a 12-h light/dark cycle with ad libitum access to food and water. The cage cleaning procedures (standard and enriched) were performed every 2 weeks. For this procedure, mice were removed and placed in holding cages for approximately 15 min. The old bedding was removed from the chamber and replaced with fresh bedding. In the enriched housing, all surfaces, toys, custom housing unit, tunnels, wheels etc. were sprayed down with 70% isopropyl alcohol and wiped clean. Once the cage was clean, test mice were placed back into the chamber. All test procedures were carried out in compliance with the National Institutes of Health Guidelines for the Care and Use of Laboratory Animals and were approved by Yale University's Institutional Animal Care and Use Committee. In addition, the reporting in the manuscript follows the recommendations in the ARRIVE guidelines.

### Environmental enrichment

This study utilized both physical and social enrichment to maximize the benefits of environmental enrichment^5^. Specifically, mice in the enriched condition were placed into a 39.5 inch-long × 26 inch-wide × 21 inch-high chamber at birth that contained a standard housing cage with bottles of water, 9 plastic toys of various sizes and textures, 3 running wheels with igloo housing, a 17 inch long clear tube, and a custom made housing unit with 2 levels made out of clear acrylic (Fig. 1b). Two litters of mice and their respective dams were placed into the chamber at a time. The two litters were of the same genotype. The mice inhabited the chamber for 25 days and were only removed for behavioral testing on PD 6 and PD 12 for vocalization assessment, PD 22 for the sensory behavior assessment, and PD 25 for the three-chamber sociability assessment (Fig. 1a) Mice in the control housing condition were raised in a standard 11.5 inch-long × 7.25 inch-wide cage with one litter per cage in a colony room. Both the enriched and standard housing conditions had a day/night cycle of 12 h and were in climate-controlled rooms set to the same temperature.

### Ultrasonic vocalizations

Ultrasonic vocalizations (USVs) were recorded on PD 6 and PD 12 as these timepoints encompass a high peak time for vocalizations for both strains and to provide a comprehensive assessment^18^. USVs were recorded using a broad-spectrum condenser microphone with a range spanning 1–125 kHz (CM16/CMPA, Avisoft Bioacoustics, Glienicke, Germany part #40011) and a recording interface (UltraSoundGate 116Hb, Avisoft Bioacoustics part # 41161/41162). The microphone was suspended above the center of the cage and vocalizations were recorded for 2 min. After the test, the pups were placed in a holding cage, returning to their home cage once testing concluded for all mice. USVs were analyzed using the DeepSqueak software freely available on Github, in accordance with prior studies^29^. Specifically, the total analysis length was set to 0, the analysis chunk length to 6, the frame overlap to 0.1 ms, the frequency low cut off to 30 kHz, the frequency high cut off to 120 kHz, and the score threshold to 0. The detection parameters were set to “high recall” to ensure that all the USVs present were detected. The files were manually processed and the signal to noise ratio optimized for each call. In addition to assessing the total number of USVs produced, we also assessed spectral and temporal characteristics, such as the duration, peak and fundamental frequency (pitch), and amplitude (loudness) of the calls, which are relevant to the ASD phenotype^15,17,18,30^.

### Somatosensory nose-poke adapted paradigm (SNAP) to assess tactile sensory preferences and repetitive exploratory behavior

Sensory and repetitive exploratory behaviors were assessed in juveniles (PD 22) using a recently developed paradigm: SNAP^22^. For this procedure, mice were individually placed on a clear elevated platform (2 inches high) that had 6, ¾ inch diameter, holes in the center. Three of the holes were lined with 80 grit coarse sandpaper (3 M Pro Grade Precision), whereas the other three holes were smooth. The holes lined with sandpaper were randomized between trials. The elevated platform was contained within a 17.5 inch-wide × 17.5 inch-long × 24 inch-high opaque, acrylic testing chamber. The mice were placed in the chamber for 5 min and were allowed to freely explore it while being video-recorded at a 20 Hz acquisition rate with a high-definition IP camera (MegaVideo AV2115DNAIv1, Arecont Vision, Glendale, CA, USA) that was mounted above the test chamber. After testing, the mice were removed from the chamber and placed into a clean holding cage. The videos were analyzed via a blinded experimenter and the number of nose pokes into the sandpaper lined holes (the rough condition) and the smooth holes (smooth condition) were recorded, as was the total quantity of nose pokes made.

### Three-chamber social interaction test

Social behavior was assessed in juveniles via the three-chamber task, as previously described^19^. Briefly, the three-chamber paradigm took place in an opaque rectangular box (60 cm-long × 44 cm-wide × 40 cm-tall) that was divided into three equal compartments that were linked via doors cut into the acrylic (44 cm-long × 20 cm-wide). The two end compartments contained an inverted wire cup (10.5 cm-tall × 10.5 cm diameter bottom × 7.6 cm diameter top, 1 cm bar spacing) while the center compartment was kept empty. The test mouse was placed in the paradigm for 10 min during a habituation trial and allowed to explore the apparatus freely. At the end of 10 min, the test mouse was removed, and a novel mouse was placed under one cup and a novel object, sized and colored like a mouse, was placed under the other cup. The test mouse was then reinserted into the apparatus for 10 min and the duration of time spent in each chamber was recorded. The location of the novel mouse and novel object were counterbalanced across trials to avoid potential confounds. The data were assessed using the ANY-maze software.

### Statistical analysis

All data were analyzed using IBM SPSS Statistics 21.0 (IBM, USA) or GraphPad Prism 7 software (La Jolla, CA). The data were split by strain and the independent variables were sex (male, female), conditions (standard, enriched), texture (rough, smooth, for the SNAP assessment) and chamber (social, middle, object, for the 3-chamber assessment). Mixed effects and univariate ANOVAs were used to analyze the data. Sidak and Tukey’s post hoc tests were used as appropriate to clarify group differences. A value of *p* < 0.05 was considered significant for each statistical test, with figures depicting the mean ± standard error of the mean (SEM).

### Supplementary Information

Supplementary Information.

## Data Availability

Raw data will be provided upon request to the corresponding author Dr. Bordey.
