# Supplementary material for: Semi-natural housing rescues social behavior and reduces repetitive exploratory behavior of BTBR autistic-like mice
Source: Sci Rep. 2023 Sep 27;13:16260. doi: 10.1038/s41598-023-43558-0 (PMC10533821; doi:10.1038/s41598-023-43558-0)
Supplement: Supplementary file 1 — Supplementary Information. [file 41598_2023_43558_MOESM1_ESM.pdf]

Table S1. ANOVA *F* and *p* values for sex effect on USV

	ANOVA analysis/ Parameters	Sex Effect	Group by Sex Interaction	Day by Sex Interaction	Day by Sex by Group Interaction
BTBR mice	Number of calls (USV)	$F_{1,36}$ = .83<br>p = .37	$F_{1,36}$ = .42<br>p = .52	$F_{1,36}$ = .32<br>p= .58	$F_{1,36}$ = .56<br>p = .46
	USV Duration	$F_{1,33}$ = .51<br>p = .48	$F_{1,33}$ = 3.75<br>p = .06	$F_{1,33}$ = .07<br>p = .80	$F_{1,33}$ = .04<br>p = .85
	USV Fundamental Frequency	$F_{1,33}$ = .00<br>p = .99	$F_{1,33}$ = 2.33<br>p = .14	$F_{1, 33}$ = .01<br>p = .91	$F_{1, 33}$ = .001<br>p =.98
	USV Peak Frequency	$F_{1,33}$ = 3.23<br>p = .08	$F_{1,33}$ = .08<br>p = .78	$F_{1,33}$ = .36<br>p = .55	$F_{1,33}$ = .73<br>p =.40
	USV Amplitude	$F_{1,33}$ = .02<br>p =.90	$F_{1,33}$ = .06<br>p =.82	$F_{1,33}$ = .41<br>p = .53	$F_{1,33}$ = 1.45<br>p = .24
B6 mice	Number of calls (USV)	$F_{1,30}$ = .03<br>p = .87	$F_{1,30}$ = 1.93<br>p = .18	$F_{1,30}$ = .36<br>p= .55	$F_{1,30}$ = 2.07<br>p = .16
	USV Duration	$F_{1,29}$ = .32<br>p = .57	$F_{1,29}$ = 2.56<br>p = .12	$F_{1,29}$ = .46<br>p = .51	$F_{1,29}$ = .77<br>p = .39
	USV Fundamental Frequency	$F_{1,29}$ = 3.98<br>p = .06	$F_{1,29}$ = 2.67<br>p = .11	$F_{1, 29}$ = .16<br>p = .69	$F_{1, 29}$ = .09<br>p =.77
	USV Peak Frequency	$F_{1,29}$ = .84<br>p = .37	$F_{1,29}$ = 3.24<br>p = .08	$F_{1,29}$ = .13<br>p = .72	$F_{1,29}$ = .003<br>p =.96
	USV Amplitude	$F_{1,29}$ = .14<br>p =.71	$F_{1,29}$ = .55<br>p = .46	$F_{1,29}$ = .08<br>p = .78	$F_{1,29}$ = .18<br>p = .68

Table S2. ANOVA *F* and *p* values for sex effect using the 3-chamber sociability task

ANOVA analysis/ Parameters	Sex Effect	Group by Sex Interaction	Chamber by Sex Interaction	Chamber by Sex by Group Interaction
BTBR Time spent in chamber with an object vs a stranger	$F_{1,35} = .09$<br>$p = .77$	$F_{1,35} = .02$<br>$p = .89$	$F_{1,35} = .27$<br>$p = .61$	$F_{1,35} = .38$<br>$p = .54$
B6 Time spent in chamber with an object vs a stranger	$F_{1,30} = .78$<br>$p = .38$	$F_{1,30} = 3.87$<br>$p = .06$	$F_{1,30} = 1.20$<br>$p = .28$	$F_{1,30} = .32$<br>$p = .58$

Table S3. ANOVA *F* and *p* values for sex effect using the Somatosensory Nose-poke Adapted Paradigm (SNAP)

ANOVA analysis/ Parameters	Sex Effect	Group by Sex Interaction	Texture by Sex Interaction	Texture by Sex by Group Interaction
BTBR Sensory preference	$F_{1,36} = .06$<br>$p = .80$	$F_{1,36} = .26$<br>$p = .61$	$F_{1,36} = .06$<br>$p = .80$	$F_{1,36} = .05$<br>$p = .82$
B6 Sensory preference	$F_{1,30} = .001$<br>$p = .97$	$F_{1,30} = .11$<br>$p = .74$	$F_{1,30} = 2.80$<br>$p = .10$	$F_{1,30} = .10$<br>$p = .76$
BTBR Number of nose pokes into smooth and sandpaper-lined holes	$F_{1,36} = 1.78$<br>$p = .19$	$F_{1,36} = 2.03$<br>$p = .16$	N/A	N/A
B6 Number of nose pokes into smooth and sandpaper-lined holes	$F_{1,35} = 1.93$<br>$p = .17$	$F_{1,35} = .92$<br>$p = .35$	N/A	N/A
